# Effects of habitat constraints on soil microbial community function

**DOI:** 10.1038/s41598-017-04485-z

**Published:** 2017-06-27

**Authors:** Naoise Nunan, Julie Leloup, Léo S. Ruamps, Valérie Pouteau, Claire Chenu

**Affiliations:** 1 iEES Paris, UMR 7618 CNRS-UPMC-INRA-IRD-Paris 7-UPEC, 4 place Jussieu, 75005 Paris, France; 2 EcoSys, 1402 UMR EGC-ECOSYS, INRA- AgroParisTech-Université Paris Saclay, 78850 Thiverval-Grignon, France

## Abstract

An underlying assumption of most soil carbon (C) dynamics models is that soil microbial communities are functionally similar; in other words, that microbial activity under given conditions is not dependent on the composition or diversity of the communities. Although a number of studies have indicated that microbial communities are not intrinsically functionally similar, most soil C dynamics models can adequately describe C dynamics without explicitly describing microbial functioning. Here, we provide a mechanistic basis for reconciling this apparent discrepancy. In a reciprocal transplant experiment, we show that the environmental context (soil and pore-network properties) of microbial communities can constrain the activity of functionally different communities to such an extent that their activities are indistinguishable. The data also suggest that when microbial activity is less constrained, the intrinsic functional differences among communities can be expressed. We conclude that soil C dynamics may depend on microbial community structure or diversity in environments where their activity is less constrained, such as the rhizosphere or the litter layer, but not in oligotrophic environments such as the mineral layers of soil.

## Introduction

An underlying assumption of soil C dynamics models is that microbial communities are all similar from a functional viewpoint, regardless of their composition or diversity. Influential soil C dynamics models such as RothC^1^ or Century^2^ for example, do not explicitly describe soil microbial communities. In these models microbial activity is represented by the decay rate constants of the various C pools, but the rate constants are not linked microbial composition, diversity or physiology. The assumption of functional similarity is central to how these models describe the responses of soil C dynamics to perturbations, such as those encountered with global climate change. Indeed, models in which the physiological responses of microbial communities to external perturbations are linked to microbial decomposition of organic C can result in different predictions of C dynamics relative to models that don’t and provide a better prediction of the apparent attenuation of the soil respiratory response to warming^3,4^. Changes in the carbon use efficiency of soil microbial communities can explain this apparent attenuation^3^ and it has been suggested that the carbon use efficiency of microbial communities is related to the communities’ diversity^5^, although this remains to be demonstrated.

However, a substantial body of literature lends credence to the assumption of functional similarity amongst microbial communities: a meta-analysis of the relationship between microbial community structure and ecosystem process rates has suggested that abiotic variables are better predictors of processes such as organic C or N mineralisation than microbial community structure^6^ and little evidence of a relationship between microbial diversity and C dynamics has been uncovered to date in mineral soils, other than when communities have extremely low levels of diversity^7–12^. The lack of relationship between process rates and microbial communities has been explained by a high degree of functional redundancy within microbial communities^13^, due to the very high levels of diversity and the physiological flexibility of microbial communities^14^. However, the studies investigating the relationship between microbial diversity and process rates established the different diversity treatments by diversity erosion using either serial dilutions of microbial suspensions^11^ or by differential fumigation^7^. These approaches reduce diversity by preferentially removing the least abundant species or the species most sensitive to fumigation. Therefore, all the diversity levels may have contained the same active microbial groups, resulting in similar levels of activity. Furthermore, significant relationships between microbial community diversity and process rates have been detected in a number of studies, although these have been in the organic or litter layers^15,16^, with low levels of diversity^16^ or the diversity effect appears to have been confounded with a biomass effect^17^. Philippot *et al*.^18^ showed a significant relationship between the diversity of denitrifiers and denitrification rates in mineral soils. However, the levels of denitrifier diversity were much lower^18^ than the levels of diversity of microorganisms involved in organic C decomposition.

A common feature of all of these studies and models is that they do not account for the microbial abiotic environment. A number of recent reviews of the literature have concluded that soil C dynamics cannot be fully apprehended without considering the constraints imposed by the abiotic environment on decomposer communities^19,20^. Although it is well established that the mineralisation of soil C is strongly related to the total organic C content of the soil, it is believed that the processes and constraints that regulate this activity occur at fine scales^21–23^. The mineralisation of organic C added to large pores has been found to be greater than in smaller pores^24,25^ and Strong *et al*.^26^ showed that the decomposition of added plant material was most strongly correlated with pores with a maximum neck diameter of 15–60 µm. However, it is now well established that microbial community structure is related to the micro-environment^27,28^ and it has been shown that there is a tight relationship between the structure of microbial communities and the pore network^25,29^: the structure and diversity of microbial communities can change as a function of the neck diameter and connectivity of the pores in which they are located. Therefore, the differences in organic C decomposition in different regions of the soil pore network cannot necessarily be attributed to abiotic constraints on microbial activity as these putative constraints are confounded with differences within the microbial communities themselves.

It is necessary to understand the mechanisms that regulate process rates and the relative importance of the different regulatory mechanisms under different conditions. The most powerful experimental approach for distinguishing the effects of microbial community properties from abiotic constraints on activity, as well as their interactions, on process rates is the “reciprocal transplant” approach, where communities from different environments are exposed to each other’s native environments, effectively decoupling the relationship between microbial communities and their environment^30^. A number of reciprocal transplant studies have been carried out. Strickland *et al*.^14^ found that microbial communities accounted for a significant portion of the variation in litter decomposition and concluded that the implicit assumption of functional equivalence in C dynamics models was incorrect for litter decay. The activity of extracellular enzymes also appeared to be dependent, at least in part, on the type of microbial community^31^. However, in both studies, the properties of the abiotic environment also explained a significant portion of the variation in activity and Griffiths *et al*.^32^ found that the resistance and resilience of microbial decomposition to external stresses was more closely related to the soil properties than to the microbial communities.

Here, we present the results of a reciprocal transplant experiment in which sterile samples of soil were inoculated with their native microbial communities or the communities from the other soil at two different matric potentials. The objective was to determine the contribution of intrinsic microbial properties and of abiotic soil properties, at both the pore and soil scales, to the variation of soil organic C mineralisation rates.

## Results

**Figure 1:**
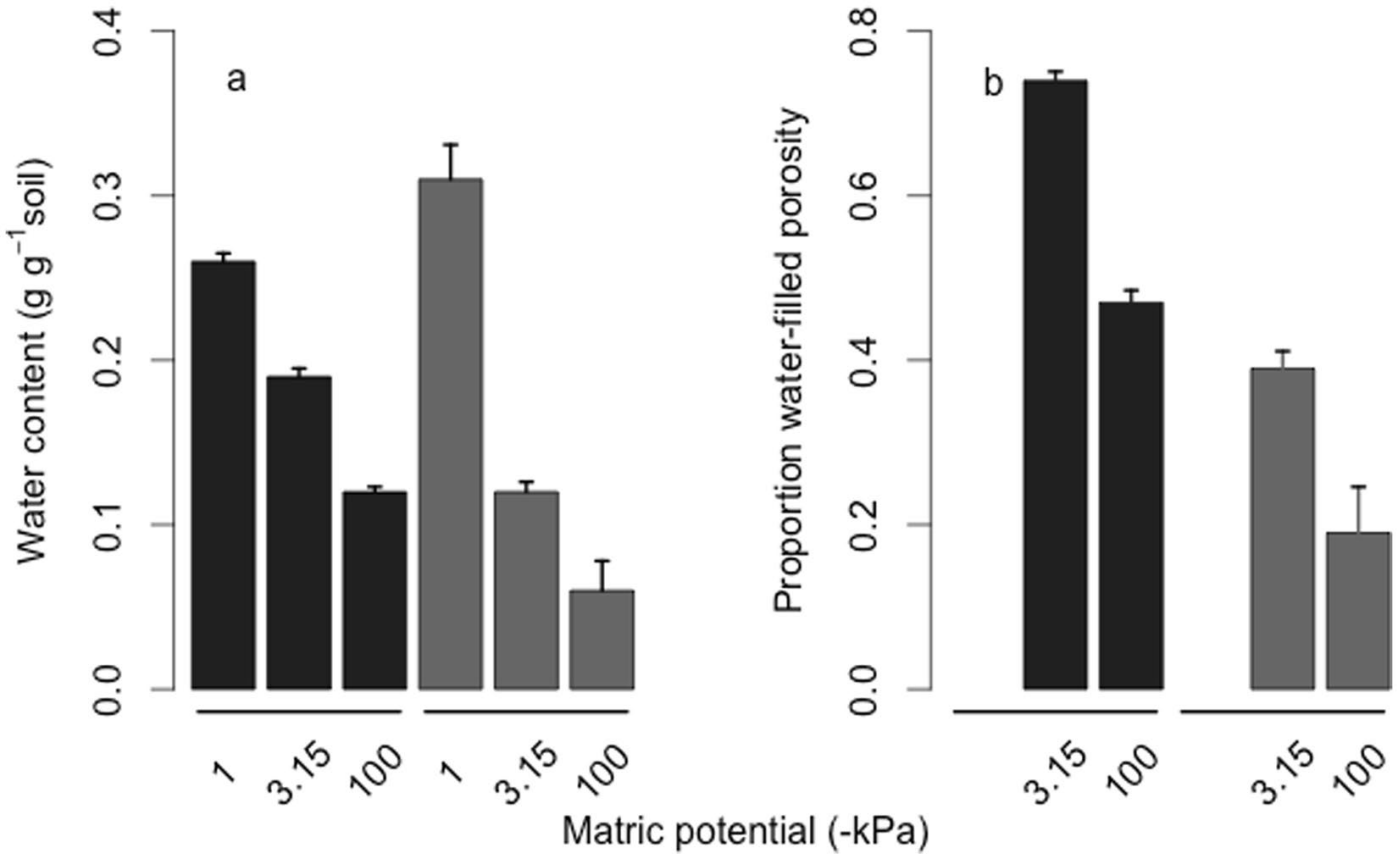
Water-filled pore volumes in the silty (black) and sandy (grey) soils at saturation and at the two incubation matric potentials (**a**), and the proportion of water-filled pores in the pore network of the silty (black) and sandy (grey) soils (**b**). Error bars are standard error of the mean (n = 4).

The silty soil retained more water at each matric potential and had a lower total porosity than the sandy soil (Fig. 1). Although the same volume of the pore network was inoculated in each treatment (0.05 cm^3^ g^−1^ soil), the proportion of the total pore volume inoculated was lower in the sandy soil (Fig. 1). The microbial inocula occupied 19 and 16% of the total pore volume in the silty and sandy soils, respectively. The water-filled pore volume represented 46 and 18% of the total pore volume in the silty and sandy soils, respectively, in the less connected treatment. In the more connected treatment, the water-filled pore volume represented 74 and 40% of the total pore volume in the silty and sandy soils, respectively (Fig. 1).

**Figure 2:**
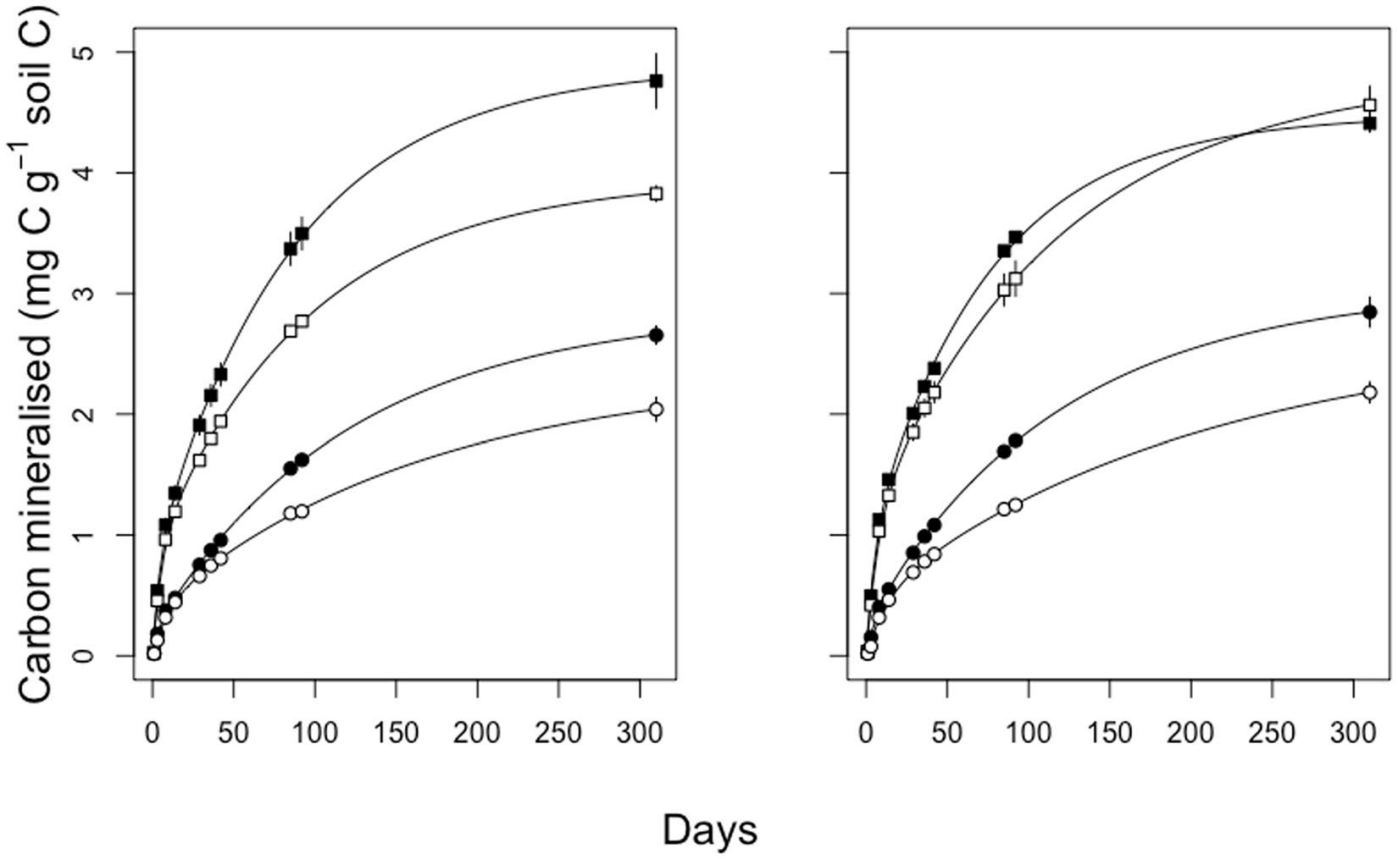
Mean carbon mineralisation curves of the microbial communities from the sandy soil (left panel) and from the silty soil (right panel) when inoculated into the silty soil (square symbols) and into the sandy soil (round symbols). Solid symbols are soils that received the microbial inoculum in the more-connected micro-environments and open symbols are soils that received the inoculum in the less-connected micro-environments. Lines represent model fit. Bars represent standard-error of the mean, where the SEM is greater than the size of the symbol.

The average cumulative mineralisation curves for each treatment combination are presented in Fig. 2. At the end of the incubation significantly more C had been mineralised in the silty soil (*P* < 0.01; Table S1, Fig. 2 and S2), regardless of the microbial community or of the micro-environment. Microbial communities in the more-connected micro-environments also produced more CO_2_ than those in less-connected micro-environments (*P* < 0.01; Table S1, Fig. 2 and S2), regardless of soil. No significant difference between the amounts of CO_2_ produced by the different microbial communities during the incubation was observed (Table S1, Fig. 2 and S2). However, there was a significant microbial inoculum x micro-environment interaction (*P* < 0.01; Table S1). This was because microbial communities from the silty soil in the more-connected micro-environment of the silty soil did not mineralise more C than when in the less-connected micro-environment of the silty soil, contrary to all the other treatment combinations, where more C was mineralised in the more-connected micro-environments (Fig. 2 and S2).

**Figure 3:**
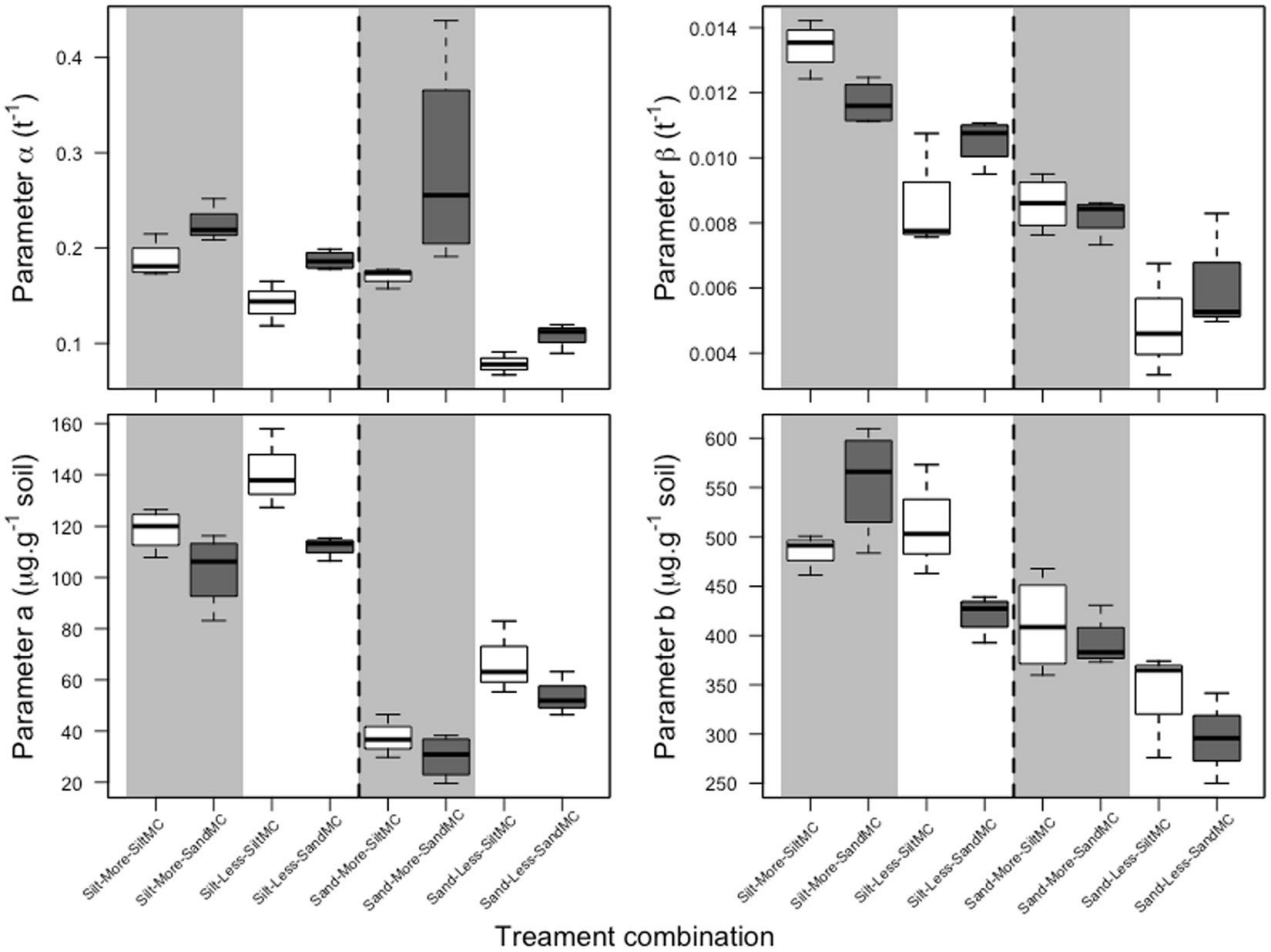
Treatment effects on the rate constant (parameter α) of the labile pool of organic C (top left panel), on the rate constant (parameter β) of the slow pool of organic C (top right panel), on the size of the labile pool of organic C (parameter a, bottom left panel) and on the size of the slow pool of organic C (parameter b, bottom right panel). The box-plots on the left of the dashed line are parameters for the silty soil (Silt) and those on the right for the sandy soil (Sand). The box-plots on the white background are the parameters for the less-connected micro-environments (Less) and those on the grey background for the more-connected micro-environments (More). The white box-plots are the parameters for microbial communities from the silty soil (SiltMC) and the dark grey box-plots for the communities from the sandy soil (SandMC).

In order to understand the effects of the different treatments in finer detail, the cumulative mineralisation curves were fitted with two-compartment first-order models and the parameters derived from these models were analysed. An ANOVA of the model parameters showed that the rate at which the labile pool of organic C was mineralised (parameter α) depended significantly on the micro-environment (*P* < 0.01) and microbial inoculum (*P* < 0.01), but not on the soil (Fig. 3 and Table S2). Although the α values associated with communities from the sandy soil were always higher than those associated with the silty soil communities, there was a small but significant microbial community x micro-environment interaction for the parameter α (Table S2). This was due to the difference between the communities being greater in the less-connected micro-environment, possibly as a result of the high variability in the measurements in one of the more-connected micro-environment treatments (Fig. 3). The size of the labile pool of organic C (parameter *a*) was significantly affected by soil type (*P* < 0.01), micro-environment (*P* < 0.01) and microbial inoculum (*P* < 0.01) (Fig. 3 and Table S3). The size of the pool of organic C that was mineralised at a slower rate (parameter *b*) and the rate at which the pool was mineralised (parameter β) were significantly affected by soil (*P* < 0.01) and micro-environment (*P* < 0.01), but not by microbial inoculum (Fig. 3 and Tables S4 and S5). There were also significant microbial inoculum x micro-environment interactions for parameters β and b. These were due to the rate constant β of the microbial communities from the sandy soil being higher than that of microbial communities from the silty soil in the less-connected micro-environment of both soils, but not in the more-connected micro-environment. The size of the C pool b associated with the microbial communities from the sandy soil was smaller than that associated with the microbial communities from the silty soil in the less-connected micro-environment of both soils but not in the more-connected micro-environments. However, the differences observed in the less-connected micro-environment were not significant when analysed on their own (*P* > 0.05).

The time taken for 99% of the labile pool of organic C to be consumed was estimated using the values of the model parameters (Table S6). Less than 1% of the pool *a* remained after 18.5 days in the sandy soil that had been inoculated in the more-connected micro-environment with the sandy microbial communities and after 60 days in the sandy soil that had also received a silty inoculum in the less-connected environment. In all the other treatment combinations the pool *a* of organic matter was 99% consumed after intermediate durations (Table S6).

**Figure 4:**
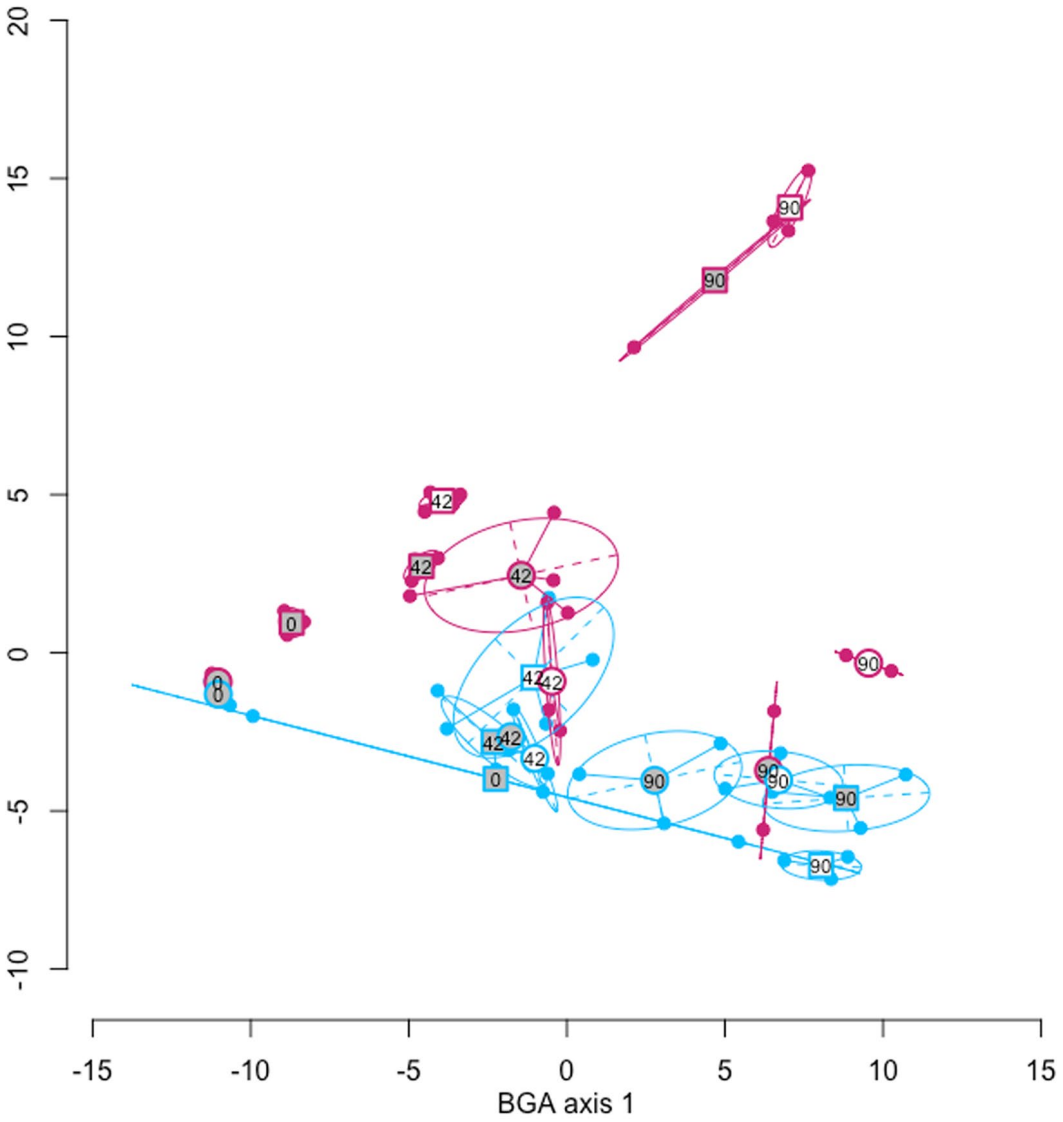
BGA ordination plot of bacterial community ARISA profiles. The large symbols indicate the group centroids and the small symbols the individual samples. The pink symbols are the microbial communities from the silty soil and the blue symbols the microbial communities from the sandy soil. The square centroid symbols indicate the silty soil and the round centroid symbols the sandy soil. The number in the centroid symbols indicate the day of analysis. The circles are envelops that incorporate 66% of the individual samples in a given group.

The structure of the microbial communities in different micro-environment x soil x day of analysis combinations was significantly different (*P* = 0.01; Fig. 4). The difference among the different communities represented 54% of the total variation in the B-ARISA data. The structure of the microbial communities in their respective native soils were the most different, whilst the communities inoculated into the non-native soil tended to converge towards the sandy communities in the sandy soil.

## Discussion

The data presented here suggest that there is a significant environmental regulatory control on C mineralisation in soil and that this control is significantly greater than the role played by the microbial communities, the effectors of the mineralisation, as others have found for enzyme production^31^ and for the resistance and resilience of microbial decomposition^32^. The relationship between the organic C content and heterotrophic respiration in soil is well established^33,34^. Here, the silty soil had higher mineralisation rates for both pools of C (Figs 2 and 3, Table S5), suggesting that the organic C of the silty soil was more mineralisable and/or that the conditions in the silty soil were more suitable for microbial activity.

At each incubation matric potential, a larger proportion of the pore network was water-filled in the silty than in the sandy soil (Fig. 1), meaning that a larger proportion of the overall resources in the silty soil were potentially available to the microbial communities, through longer solute and enzyme diffusion pathways, to the microbial communities^23,35^. Assuming that the organic C was homogeneously distributed in the soils and that all the organic C in the water-connected pore space was potentially available to the microbial communities, then 6.21 and 2.65 mg C g^−1^ soil (total organic C x proportion of pore-network that was water-filled) were potentially available to the microbial communities in the more-connected micro-environment in the silty and sandy soils, respectively. The amounts of C mineralised during the incubation were therefore equivalent to 6.2 and 6.9% of the potentially available organic in the silty and sandy soils, respectively. The same calculation for the less-connected micro-environment treatment shows that 8.8 and 11.2% of the potentially available organic C in the silty and sandy soils, respectively, were mineralised during the incubation. Were the assumptions made here correct, then, despite the large differences in total C mineralisation in the two soils (Fig. 2), the proportion of available C mineralised was similar, suggesting that it is not necessary to invoke microbial community properties or organic matter composition in order to accurately describe C mineralisation; microbial access to organic C may suffice. Of course, the assumption that organic C is homogeneously distributed in soil is unlikely to be true^36,37^. Furthermore, all the C in the water-connected pore space is unlikely to be accessible to the microbial communities, particularly in the silty soil. The greater surface area of the silty soil, due to the higher clay content, is likely to have resulted in lower water film thicknesses and therefore longer solute diffusion pathways^38^, which may have reduced the potential availability of the organic C. The higher clay content may also have resulted in a higher proportion of the organic matter being adsorbed and hence unavailable to microorganisms^21^. Nevertheless, mapping the availability of organic C to microbial communities may be useful for mechanistic models of soil organic C mineralisation. This would require that the spatial distribution of organic C in relation to connected diffusion pathways to microbial communities at different matric potentials be accounted for.

Differences in C mineralisation may also have arisen because of an incomplete colonisation of the targeted micro-environments, especially in the case of the less-connected micro-environment. If the more connected micro-environment was more widely colonised than the less-connected micro-environment, then one would expect higher mineralisation rates because a higher probability of encounter between microorganisms and substrate^39–41^. The relationship between matric potential and soil respiration is also believed to be influenced by osmotic stress, gas diffusion and the availability of O_2_^35^. However, the matric potentials used in this study are unlikely to have induced any significant osmotic stress on the microbial communities^42^.

The two microbial communities used in this study were not intrinsically functionally similar. At the beginning of the incubation, when the rate at which the labile pool of organic matter was mineralised was very high, the microbial communities showed significant differences both in the rates at which they mineralised the available organic C (differences in the parameter α) and in their capacities to utilise the range of molecular types available as suggested by the differences in the sizes of the labile organic C pool *a*. The rate at which the labile pool of organic C was mineralised was an order of magnitude greater than the rates that are generally found in bulk mineral soils^43,44^ but similar to rates found for added labile organic matter^22^. This suggests that the differences between the microbial communities were expressed when microbial activity was higher than is generally found in bulk soils and that the activity was higher because of a high availability of organic substrate, possibly made available during the sterilisation process^45^.

As the incubation progressed however, the differences in functioning between the two communities diminished (i.e. neither of the parameters related to the mineralisation of the organic C pool *b* were significantly related to the microbial communities). Two studies have shown that the composition of complex microbial inocula that are initially different tend to converge when placed in the same soil environment^31,32^, which might explain the convergence in functioning of the different communities^30^. This was not the case here however, as the differences among communities did not converge. In fact, the communities from the silty soil that were inoculated into the silty soil tended to diverge during the incubation (Fig. 4). Therefore, even though the functioning of the communities converged after the readily mineralisable pool of organic C had been consumed, the composition of the microbial communities did not.

Despite the fact that the composition of the communities remained distinct, it is plausible that the active component of the two communities became more similar during the incubation, resulting in a similar mineralisation activity. There may also have been what has been called a “portfolio effect”^46^, where the sum of the different individual activities contained in the communities was the same, regardless of the communities’ compositions. Although plausible, neither of these explanations are likely, as they would require that the active component of the two communities converge in two different soils or that the sum of the constituent activities of the microbial communities be similar in the two different soils.

The mineralization rates of the two microbial communities converged at rates that were an order of magnitude below the mineralization rates of the labile pool of organic C (parameter β versus parameter α; Fig. 3; Table S6) and therefore at rates well below what both communities were capable of. It is possible that the low mineralisation rates at which the communities converged were due to the remaining available organic C being of a complex nature, that required the production of a broad spectrum of enzymes to be produced for decomposition to proceed, thus resulting in similar low mineralisation rates for both communities. However, a number of recent studies have suggested that stable C in soil is not chemically recalcitrant but tends to be composed of relatively simple molecular types^47,48^ that are protected by adsorption to minerals or are inaccessible to microbial degraders.

An equally plausible explanation for the convergence of mineralisation rates is that it occurred due to the environment constraining the intrinsic differences that existed between the communities. The dramatic difference in mineralisation rates of the two organic C pools suggests that constraints on activity during the latter part of the incubation were so great that the manifest differences between the microbial communities’ functioning could no longer be expressed. Were this scenario to be true, then the functional redundancy that has often been attributed to microbial communities may, in fact, be the result of environmental constraints: some form of habitat or environment-induced functional equivalence in soil microbial communities. The nature of the constraint or constraints cannot be ascertained, however, it is appealing to suggest that low levels of access to organic substrate constrained the mineralisation activity of the microbial communities. Studies that have identified a relationship between microbial communities and C mineralisation tend to have been carried out in the litter or organic layers of soil^14–16^, whilst those that have not have been carried out in mineral soil^8,9,11^, with lower levels of organic C present.

Habitat filtering is known to be a dominant structuring agent in microbial communities^49^ and other studies that have carried out reciprocal transplant experiments in mineral soil have shown that the structure of microbial communities is principally governed by the soil environment in which they are resident^31,32^. Here, although the structures of the microbial communities inoculated into the sandy soil were similar, this was not the case for the communities inoculated into the silty soil (Fig. 4). It is known that the pH of soil is one the primary drivers of microbial community structure^50^. The pH of the sandy soil was lower than that of the silty soil (5.2 vs 6.8). These data suggest therefore, that microbial communities from the low pH sandy soil were more resilient to change or that the acidic environment of the sandy soil exerted more pressure on the microbial communities.

We further showed that the micro-environment, in the form of the connectivity of the local environment, also affected the structure of the microbial communities, albeit to a much lesser extent. This tends to corroborate the conclusion drawn in Ruamps *et al*.^25^ that there is a pore scale microbial biogeography. The differences among treatments and dates accounted for 54% of the total variability in the B-ARISA profiles and the two axes presented in Fig. 4 only 16% of the total variability. Other BGA axes also show differences among treatments and dates (data not shown). Furthermore, due to stochastic processes that can occur during the inoculation and the rapid growth stages at the beginning of the incubation, high inter-replicate variability can be expected in this type of experiment^8,11^.

## Conclusions

The key take-home messages that emerge from this study are: (1) that microbial respiration in soil may be dependent on the spatial organisation and connectivity of diffusion pathways between substrate and decomposers and (2) that the relationship between microbial communities and soil C dynamics may be dependent on trophic conditions. This is one of the first empirical studies to indicate that the relative contribution of species to community functioning changes with environmental context^51^. In environments were the activity of microbial communities is not as restricted as in bulk mineral soil, such as in the litter or organic layers or the rhizosphere, C mineralisation may possibly be related to the composition or diversity of the resident microbial communities. However, when microbial activity is restricted to levels well below the rates at which the communities can metabolise, as might be the case in mineral soil layers, then the composition or diversity of the microbial communities may no longer be consequential because all communities mineralise at the rate at which the abiotic constraints allow. This scenario provides an elegant mechanistic basis for soil C dynamics models.

## Materials and Methods

### Soil sampling and biocidal treatment

Soil was collected from the surface 10 cm at two sites in Ile de France region (France). Samples were taken from plots under wheat at the “Closeaux” field experiment (INRA research centre in Versailles, France) and from a natural grassland at the CEREEP (Centre de Recherche en Écologie Expérimentale et Prédictive) experimental station (Saint-Pierre-lès-Nemours, France). The Closeaux soil is classified as an Eutric Cambisol (17.4% clay, 53% silt and 29.6% sand) with a pH of 6.8, and organic C and total N contents of 13.5 and 1.23 g.kg^−1^ soil, respectively. The CEREEP soil is classified as a Sandy Cambisol (6.9% clay, 19% silt and 74.1% sand). The pH of the soil was 5.2 and the organic C and total N contents were 14.7 and 1.19 g.kg^−1^, respectively. The soils were sieved (<5 mm) to remove stones and plants residues. The soils are referred to as silty (Silty Eutric Cambisol) and sandy (Sandy Cambisol) in the following. The soils had different pore size distributions, as demonstrated by the measured moisture release curves (Fig. S1). The choice of two soils with similar C contents but differing in many other respects was deliberate, as there is a well established relationship between total C and the mineralisation rates of organic C in soil. This relationship *per se* was not the object of this study, hence the choice of soils with similar total C contents.

The soils were sterilised using gamma-irradiation as it is an effective biocidal treatment, but does not disrupt the physical structure of soil nor have a dramatic effect on non-biomass organic matter^52^. It has been shown that gamma irradiation affects primarily molecules of biomass origin^53^. The soil was air-dried to a matric potential of −1000 kPa and then sealed in polypropylene bags for sterilisation by gamma-irradiation (IONISOS, ZA). The soil was irradiated in a relatively dry state to minimise the impact of irradiation on the chemical stability of soil organic matter^52^. The soil (20 kg) was exposed to a 70 kGy dose (with a minimum guaranteed exposure of 45 kGy). After sterilisation, the soil was placed at 4 °C for 15 days to ensure that the free radicals that may have been generated during gamma irradiation had disappeared prior to the start of the incubation.

### Extraction of microbial communities

Microbial communities from both soils (silty-MC and sandy-MC) were extracted from non-sterile samples by adding 200 mL ¼ strength Ringer’s solution to 20 g soil and shaking the suspension with glass beads for 30 minutes. The soil suspension was left to settle for 10 minutes and the supernatant, containing the microbial suspension, was removed and used to inoculate the sterilized soils.

### Moisture retention curves

The moisture retention curves of the soils were established in triplicate using Richard’s pressure plates (Fig. S1). The water volume at saturation was considered to represent the total pore volume in the soils. The volume of water in the soils at the incubation matric potentials (see below) represented the water-filled volume during the incubation of the soils. The percentage of the total pore volume occupied by the microbial inocula was determined by dividing the volume of the microbial suspension used for inoculation (0.045 cm^3^ g^−1^ soil) by the total pore volume.

### Microbial inoculation of sterilized soil

Sterilized samples of the silty and the sandy were inoculated with either their native microbial suspensions or with microbial suspensions from the other soil. The soils were inoculated at two different matric potentials in order to place the microbial communities in distinct regions of the sterilized soils’ pore networks, as described previously^54–56^. Half the samples were equilibrated at a matric potential below −1600kPa and brought to a matric potential of −100kPa with the microbial suspensions whilst the other half were equilibrated at a matric potential slighlty above −100kPa and brought to −3.15kPa with the microbial suspensions. The microbial communities in the first half of the samples were more likely to be found in pores or crevices with a neck diameter <3 µm or along surfaces of larger pores where the water-film thickness was not sufficient to completely immerse a bacterial cell^23^. The microbial communities in the second half of the samples were more likely to have been placed in pores with a maximum neck diameter ranging from 6 to 97 µm and were more likely to have been immersed in thicker water-films with longer, more connected diffusion pathways. The microbial communities in the first half of the samples were therefore located in a physical micro-environment in which the diffusion pathways, necessary for substrate and enzymes to come into contact and, ultimately, for microbial activity, were less connected^23,57^. For simplicity, these two treatments are referred to as “less-connected micro-environment” and “more-connected micro-environment” in the following, although any effects of this treatment on microbial respiration were likely due to more than just the connectivity of the diffusion pathways, such as the local chemical environment and O_2_ availability. The same volume of microbial suspension (4.5 mL) was added to each 90 g sterile soil aliquot in all treatments. The same volume of inoculant was used to ensure that the same volume of pore space was inoculated in all treatments. There were 8 treatment combinations (2 soils x 2 communities x 2 micro-environments) and four replicates per treatment combination, resulting in 32 microcosms being prepared for incubation (see next section). In order to accommodate the destructive sampling necessary for the microbial structure analyses, 4 subs-samples (90 g each) were prepared per replicate. Microbial community structure was determined in 1 of the sub-samples per replicate 6 h after inoculation and the other sub-samples were incubated as described below.

### Incubation and respiration measurements

The inoculated samples (3 × 90 g) were incubated in 1 L jars fitted with a septum for headspace sampling. All the incubation equipment was autoclaved prior to the addition of the soil samples. The samples were incubated at matric potentials of −3.15 kPa (more-connected micro-environment) or −100 kPa (less-connected micro-environment) at 20 °C in the dark for 310 days. The production of CO_2_ was measured 10 times (days 1, 3, 8, 14, 29, 36, 42, 85, 92 and 310) during the incubation by gas chromatography (Agilent 3000 A, Massy, France). The microcosms were flushed under sterile conditions if the headspace CO_2_ concentration reached 2% by leaving the jars open under a sterile laminar flow hood for 2 hours. The CO_2_ concentration was measured again after flushing. The moisture content of the samples was verified gravimetrically and corrected by adding filter-sterilised water when necessary.

### Microbial community structure analysis

Microbial community structure was analysed on days 0, 42 and 92 using a bacterial automated rRNA intergenic spacer analysis (ARISA) fingerprinting approach based on the length polymorphism of the bacterial InterGenic Spacer (IGS)^58^ (GenoSol platform). It has been shown that ARISA fingerprinting has as much explanatory power of process rates as next generation sequencing technology^6^.

Total nucleic acids were extracted from 0.5 g soil samples (dry weight equivalent) with the FastDNA Spin kit for soil in combination with the FastPrep FP120 bead beating system (MP-Biomedicals, CA, USA), according to the manufacturer’s instructions. Bulk total DNA was purified by elution through Geneclean Turbo columns according to the manufacturer’s instructions (MP Biomedicals, CA, USA). The concentration and purity of the resulting DNA were determined spectrophotometrically by measuring the absorbance at 260 and 280 nm and calculating the ratio A260/A280 (NanoDrop ND-1000 spectrophotometer).

The bacterial intergenic spacers were amplified with the primer set: S-D-Bact-1522-b-S-20/ L-D-Bact-132-a-A-18, with 50 ng of DNA as template^58^. The S-D-Bact-1522-b-S-20 primer was labeled at the 5’-end with the IRD800 dye fluorochrome (MWG SA Biotech, Ebersberg, Deutschland) to allow for the detection of the PCR fragments by the LiCor DNA sequencer system (ScienceTec, Les Ulis, France). Data obtained from the 1D-Scan software (Sciencetec) were converted into a table summarizing band presence (that is, peaks) and intensity (that is, height or area of peak) using the PrepRISA program^58^; 100 peaks, 2 bp resolution and Gaussian peak area were used to provide a robust analysis of bacterial communities. The resulting bacterial-ARISA data matrix (bacterial communities as rows and bands as columns) accounted for the presence/absence and relative intensity of bands.

### Statistical analysis and modelling

The cumulative respiration curves were described by fitting a two-compartment first-order model (Eq. 1) to the experimental data using a modification of the Levenberg-Marquardt nonlinear least-squares algorithm in the R minpack.lm package^59^:

1 $$C{O}_{2}=a({\rm{1}}-{e}^{-\alpha t})+b({\rm{1}}-{e}^{-\beta t})$$

were *a* is a pool of organic C that decomposes rapidly, *b* is an organic C pool that decomposes at a slower rate and *α* and *β* are the respective decomposition rates. The two-compartment first-order model provided a good fit to the respiration curves because the process of sterilisation releases readily mineralisable C, primarily of microbial origin, into the soil solution, which results in a rapid respiratory flush as the microbial inocula colonise the dead biomass^45,55^. This is the basis for the fumigation-incubation measurement of the soil microbial biomass^45^. The pool *b* of organic C can be considered to represent the organic matter unaffected by the biocidal treatment.

In order to determine the effects of soil, micro-environment (matric potential) or microbial community origin on microbial community structure, a between-group analysis (BGA) was carried out on the B-ARISA profiles using the ade4 package^60^ in the R environment (Studio Team, 2015; RStudio: Integrated Development for R. RStudio, Inc., Boston, MA. URL http://www.rstudio.com/). BGA is a supervised classification method that is based on carrying out an ordination of groups of samples rather than of samples. Whilst principal component analysis (PCA) maximises the variance amongst individual samples, BGA maximises the differences among the centroids of groups of samples. The individual sample locations are subsequently projected onto the resulting axes. The B-ARISA profiles were grouped according to treatment combination and day of analysis. The ARISA profiles were transformed using the Hellinger distance prior to carrying out the BGA^61^.

### Data availability statement

The datasets generated during the current study are available from the corresponding author on reasonable request.

## Electronic supplementary material

Supplementary Information
