# Author Correction: Effects of habitat constraints on soil microbial community function

**DOI:** 10.1038/s41598-018-22119-w

**Published:** 2018-03-01

**Authors:** Naoise Nunan, Julie Leloup, Léo S. Ruamps, Valérie Pouteau, Claire Chenu

**Affiliations:** 1 iEES Paris, UMR 7618 CNRS-UPMC-INRA-IRD-Paris 7-UPEC, 4 place Jussieu, 75005 Paris, France; 2 EcoSys, 1402 UMR EGC-ECOSYS, INRA- AgroParisTech-Université Paris Saclay, 78850 Thiverval-Grignon, France

Correction to: *Scientific Reports* 10.1038/s41598-017-04485-z, published online 27 June 2017

The Acknowledgements section in this Article is incomplete.

“This work was funded by the Agence Nationale de la Recherche – project numbers: ANR-09-SYSC-007 and ANR-15-CE01-006-03.”

should read:

“This work was funded by the Agence Nationale de la Recherche – project numbers: ANR-09-SYSC-007 and ANR-15-CE01-006-03. The ARISA analyses were carried out at the GenoSol analytical platform, Dijon, France.”

