# Supplementary material for: Effects of habitat constraints on soil microbial community function
Source: Sci Rep. 2017 Jun 27;7:4280. doi: 10.1038/s41598-017-04485-z (PMC5487364; doi:10.1038/s41598-017-04485-z)
Supplement: Supplementary file 1 — Supplementary Information [file 41598_2017_4485_MOESM1_ESM.docx]

**Effects of habitat constraints on soil microbial community function**

Naoise Nunan^1^*, Julie Leloup^1^, Léo S. Ruamps^1^, Valérie Pouteau^2^, Claire Chenu^2^

^1^ iEES Paris, UMR 7618 CNRS-UPMC-INRA-IRD-Paris 7-UPEC, 4 place Jussieu, 75005 Paris, France

^2^ EcoSys, 1402 UMR EGC-ECOSYS, INRA- AgroParisTech-Université Paris Saclay, 78850 Thiverval-Grignon, France

*Corresponding Author: Naoise Nunan; naoise.nunan@upmc.fr


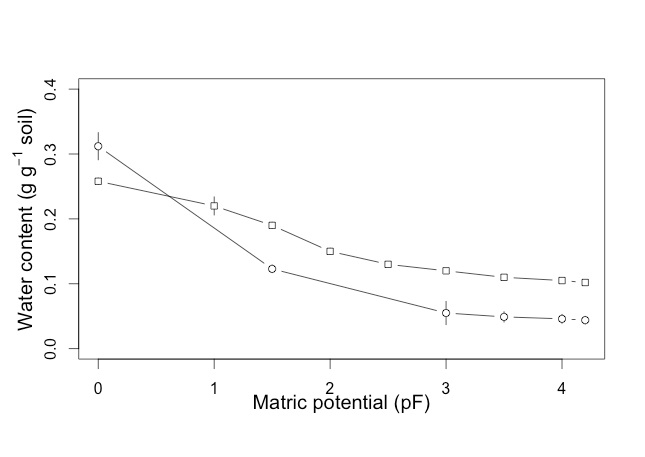
Fig S1 Water retention curves for silty (□) and sandy (○) soils. Bars represent standard error of the mean (n=3), where error is larger than the size of the symbol.


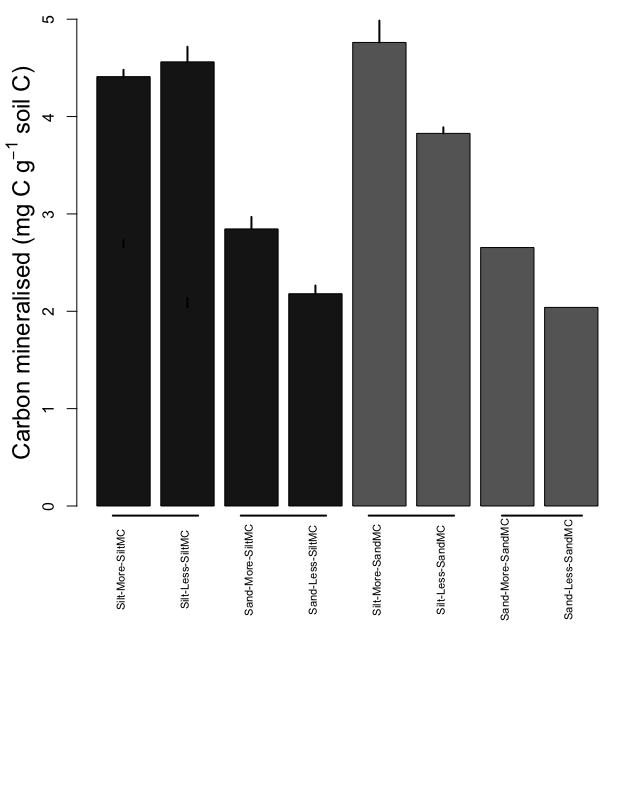


Fig S2 Amount of organic C mineralised during the incubation. Black bars are for microbial communities from the silty soil and grey bars are for microbial communities from the sandy soil. Bars represent standard error of the mean (n=4).

Table S1 ANOVA table for effects of soil x micro-environment x microbial community on the total amount of organic C mineralised.

| Source of variation | df | Sum Sq | Mean Sq | F-value | *P* |
| --- | --- | --- | --- | --- | --- |
| **Soil** | **1** | **26.745** | **26.745** | **463.909** | **8.48 10^-16^** |
| **Mico-Environment** | **1** | **1.543** | **1.543** | **26.765** | **3.98 10^-05^** |
| Microbial Community | 1 | 0.175 | 0.175 | 3.032 | 0.09627 |
| Soil x Micro-Environment | 1 | 0.077 | 0.077 | 1.340 | 0.26007 |
| Soil x MC | 1 | 0.003 | 0.003 | 0.044 | 0.83675 |
| **Micro-Environment x MC** | **1** | **0.514** | **0.514** | **8.924** | **0.00702** |
| **Soil x Micro-Environment x MC** | **1** | **0.575** | **0.575** | **9.966** | **0.00476** |
| Residuals | 21 | 1.211 | 0.058 |  |  |

Table S2 ANOVA table for effects of soil x environment x microbial community on the rate constant of the labile pool of organic C (parameter α).

| Source of variation | df | Sum Sq | Mean Sq | F-value | *P* |
| --- | --- | --- | --- | --- | --- |
| Soil | 1 | 0.00240 | 0.00240 | 1.216 | 0.28271 |
| **Micro-Environment** | **1** | **0.05101** | **0.05101** | **25.817** | **4.95 10^-05^** |
| **Microbial Community** | **1** | **0.02623** | **0.02623** | **13.279** | **0.00152** |
| **Soil x Micro-Environment** | **1** | **0.01538** | **0.01538** | **7.786** | **0.01097** |
| Soil x MC | 1 | 0.00246 | 0.00246 | 1.246 | 0.27687 |
| Micro-Environment x MC | 1 | 0.00251 | 0.00251 | 1.273 | 0.27198 |
| Soil x Micro-Environment x MC | 1 | 0.00387 | 0.00387 | 1.959 | 0.17624 |
| Residuals | 21 | 0.04149 | 0.00198 |  |  |

Table S3 ANOVA table for effects of soil x environment x microbial community on the size of the labile pool of organic C (parameter a).

| Source of variation | df | Sum Sq | Mean Sq | F-value | *P* |
| --- | --- | --- | --- | --- | --- |
| **Soil** | **1** | **37581** | **37581** | **347.870** | **1.51 x 10^-15^** |
| **Micro-Environment** | **1** | **2862** | **2862** | **26.495** | **4.24 x 10^-05^** |
| **Microbial Community** | **1** | **1914** | **1914** | **17.714** | **0.000394** |
| Soil x Micro-Environment | 1 | 257 | 257 | 2.378 | 0.138015 |
| Soil x MC | 1 | 252 | 252 | 2.333 | 0.141615 |
| Micro-Environment x MC | 1 | 168 | 168 | 1.559 | 0.225594 |
| Soil x Micro-Environment x MC | 1 | 25 | 25 | 0.235 | 0.632909 |
| Residuals | 21 | 2269 | 108 |  |  |

Table S4 ANOVA table for effects of soil x environment x microbial community on the rate constant of the slow pool of organic C (parameter β).

| Source of variation | df | Sum Sq | Mean Sq | F-value | *P* |
| --- | --- | --- | --- | --- | --- |
| **Soil** | **1** | **1.203 10^-04^** | **1.203 10^-04^** | **94.370** | **3.21 10^-09^** |
| **Micro-Environment** | **1** | **5.78210^-05^** | **5.78210^-05^** | **45.360** | **1.16 10^-06^** |
| Microbial Community | 1 | 9.00010^-08^ | 9.00010^-08^ | 0.074 | 0.78876 |
| Soil x Micro-Environment | 1 | 0 | 0 | 0.001 | 0.97840 |
| Soil x MC | 1 | 3.20010^-07^ | 3.20010^-07^ | 0.248 | 0.62343 |
| **Micro-Environment x MC** | **1** | **1.25110^-05^** | **1.25110^-05^** | **9.816** | **0.00503** |
| Soil x Micro-Environment x MC | 1 | 1.61010^-06^ | 1.61010^-06^ | 1.262 | 0.27398 |
| Residuals | 21 | 2.67710^-05^ | 1.27010^-06^ |  |  |

Table S5 ANOVA table for effects of soil x environment x microbial community on the size of the slow pool of organic C (parameter b).

| Source of variation | df | Sum Sq | Mean Sq | F-value | *P* |
| --- | --- | --- | --- | --- | --- |
| **Soil** | **1** | **117896** | **117896** | **67.552** | **5.33 x 10^-8^** |
| **Micro-Environment** | **1** | **37267** | **37267** | **21.353** | **0.000147** |
| Microbial Community | 1 | 1838 | 1838 | 1.053 | 0.316442 |
| Soil x Micro-Environment | 1 | 1159 | 1159 | 0.664 | 0.424214 |
| Soil x MC | 1 | 1075 | 1075 | 0.616 | 0.441347 |
| **Micro-Environment x MC** | **1** | **16108** | **16108** | **9.229** | **0.006254** |
| **Soil x Micro-Environment x MC** | **1** | **8430** | **8430** | **4.830** | **0.039323** |
| Residuals | 21 | 36650 | 1745 |  |  |

Table S6 Parameters derived from model fits to cumulative respiration curves (average of four replicates and standard error of the mean) and days until 99% labile pool consumed.

| Soil | Micro-environment | Inoculum | α | a | ß | b | days |
| --- | --- | --- | --- | --- | --- | --- | --- |
| Silty | More-connected | Slity-MC | 0.19±0.01 | 118±4 | 0.013±0.000 | 486±8 | 25.25 |
|  |  | Sandy-MC | 0.23±0.01 | 103±7 | 0.012±0.000 | 556±27 | 21.25 |
|  |  |  |  |  |  |  |  |
|  | Less-connected | Slity-MC | 0.14±0.01 | 141±9 | 0.009±0.001 | 513±32 | 33.0 |
|  |  | Sandy-MC | 0.19±0.01 | 112±2 | 0.011±0.000 | 421±10 | 25.25 |
|  |  |  |  |  |  |  |  |
| Sandy | More-connected | Slity-MC | 0.17±0.01 | 37.4±3.4 | 0.009±0.000 | 411±25 | 27.5 |
|  |  | Sandy-MC | 0.29±0.06 | 29.9±4.3 | 0.008±0.000 | 392±13 | 18.5 |
|  |  |  |  |  |  |  |  |
|  | Less-connected | Slity-MC | 0.08±0.01 | 67.1±8.2 | 0.013±0.000 | 336±31 | 60.0 |
|  |  | Sandy-MC | 0.11±0.01 | 53.8±5.0 | 0.013±0.000 | 295±27 | 44.0 |
